# A Case of Monstrosity

**Published:** 1884-03

**Authors:** C. Hard

**Affiliations:** Ottawa, Illinois


### ARTICLE V.

### A CASE OF MONSTROSITY. A CHILD WITH A DOG'S HEAD.

By C. HARD, M.D., Ottawa, Illinois.

On the night of September 8, 1883, I was called to the vicinity of Starved Rock, in this county, to attend Mrs. G., a German woman, in her second labor. The waters had been discharged several hours before I arrived. The pains were violent and frequent. On examination, I found the os only partially dilated. A soft mass presented, which at first I supposed to be a breech presentation. The pains continued almost without cessation for hours, when, just at sunrise, she was delivered of a very large female child, weighing $14\frac{1}{2}$ lbs. The body was well-formed and perfect, but the head was almost the exact counterpart of a bull pup, and was the most hideous monstrosity I ever saw. The eyes were high up on the forehead, large and round, and protuberant, and were two and a half inches apart; no eyebrows. The nose was long and flat, and continued to the mouth, with wide open nostrils; the distance from forehead to nose and mouth 3½ inches. The ears were small and long, standing out from the sides of the head one inch, and looked like the cropped ears of a stable dog; they were 3½ inches apart. Back of the ears was a single tuft of coarse reddish hair, about one inch long. There was no back part to the head; or rather, no bones, but a simple sac containing a soft, semi-fluid mass. Taking the whole face together, the resemblance to a dog was most striking, and was at once remarked upon by all present, some of whom were anxious that I should despatch it at once, but I allayed their fears by assuring them that the child would not live. It did live four hours, giving out faint moans from time to time.

Owing to the sensitiveness of the parents, I was unable to procure the head, even for the purpose of making a cast. The husband, upon questioning the mother, who does not speak English, gave me the following statement: Her father kept a large dog chained to his kennel, and during the early part of her pregnancy, she was in the habit of visiting at his house. While going past the dog one evening he suddenly sprang at her, barking furiously, and frightened her almost out of her senses, and she had labored under the impression ever since that her child would be marked. Such is their story, and I give it for what it is worth. The parents are both well-formed, and have one child two years old, also well-formed and bright, and there were no ties of consanguinity between them. What caused the monstrosity?

---

Manaca, the root of Francisca uniflora acts energetically on the lymphatic system. It is used largely in Brazil as an antisyphilitic remedy and as a purgative and diuretic. In rheumatism it seems to be a specific and it is said that twenty drops of the fluid extract given twice daily are sufficient to suppress the severest attack. In Brazil it is called *mercurio vegetal.*

